# Factors associated with burnout among Chinese nurses during COVID-19 epidemic: a cross-sectional study

**DOI:** 10.1186/s12912-022-00831-3

**Published:** 2022-02-28

**Authors:** Zhiying Wan, Mengfei Lian, Hui Ma, Zhongxiang Cai, Yunyan Xianyu

**Affiliations:** 1 Department of Psychiatry, Renmin Hospital of Wuhan University, Wuhan, China; 2 Department of Nursing, Renmin Hospital of Wuhan University, Wuhan, China; 3 Cardiovascular Medicine Department, Renmin Hospital of Wuhan University, Wuhan, China

**Keywords:** Burnout, Anxiety, Nurses, COVID-19

## Abstract

**Background:**

The coronavirus disease 2019 (COVID-19) epidemic has broken out and even spread globally. The healthcare system worldwide faces enormous challenges, and nurses are at the highest risk as one of the leading forces. It's worth paying attention to nurses' anxiety and job burnout. This study aimed to investigate nurses' levels of burnout and anxiety during the epidemic of COVID-19 and to analyze influencing factors of burnout.

**Methods:**

A cross-sectional survey was conducted from 19 to 25 February 2020. Questionnaires such as the basic information questionnaire, Maslach Burnout Inventory-General Survey (MBI-GS), and State-Trait Anxiety Inventory (STAI) were used among 1011 nurses in Wuhan tertiary hospitals via the online survey. The final number of valid questionnaires was 885. The effective response rate was 87.5%.

**Results:**

The average score of MBI-GS was 11.50, 6.02, 24.47, respectively. The average score for state anxiety was 45.52 and trait anxiety, 43.78. Anxiety was positively associated with emotional exhaustion and cynicism, and negatively related to personal accomplishment. The protective factors of burnout were personnel agency, five years or less work experience, living in hospital dormitory, Wuhan medical team, working time exceeding 9 h, and the best knowledge of COVID-19. The absence of siblings, median job title, working in isolation wards, three or more night shifts per week, living in hotels, and being surrounded by confirmed or suspected medical staff were all negative factors.

**Conclusions:**

Nurses had high anxiety levels during the COVID-19 period, but the level of burnout was mild to moderate. Managers should continue to pay attention to nurses' psychological state and related factors and intervene to stabilize the nursing team.

## Background

In December 2019, Novel coronavirus pneumonia began erupting in Wuhan, Hubei Province, as the quake's epicenter. As the epidemic has developed, it has spread around the world. Since the outbreak of the COVID-19, China's national and local government leaders have worked together to take a series of effective prevention and control measures. On 23 January 2020, Wuhan City COVID-19 Prevention and Control Center issued a circular requesting the city to suspend operations and cut off transmission lines [1]. Hospitals play an irreplaceable role in prevention and treatment as effective treatment institutions. As of 28 February, more than 90 national and provincial medical teams, about 13,000 senior healthcare workers had gathered in Wuhan to work with local medical staff to treat patients [2].

Nurses are the most significant part of the healthcare professionals, and they have played an extraordinary role in combating COVID-19. During the pandemic, especially the initial stage, nurses struggled with insufficient resources, intensive work, and uncertainty. In their daily work, they witness the pain and suffering of the patients [3], wear cumbersome personal protective equipment (PPE), and perform tedious and detailed care for confirmed patients in isolated areas. In addition, they are at a higher risk of contracting the virus than others. Because of the possibility of transmitting the virus to families and traffic restrictions, most medical personnel could not return home and had to stay in hospital dormitories and hotels. Therefore, facing such high levels of psychological pressure and work-related stress, they experience mental health problems such as depression and anxiety [4] and experience fatigue, burnout, and even consider quitting their jobs [5, 6].

Burnout is a state of mind caused by chronic emotional or psychological stress [7]. Maslach described this condition as emotional exhaustion (the state of being emotionally drained), depersonalization (the loss of compassion and concern), and a diminished sense of personal accomplishment at work [8, 9]. On 28 May 2019, the World Health Organization (WHO) declared in the 11th revision of the International Classification of Diseases (ICD-11) that burnout is a "professional phenomenon", a chronic workspace stress syndrome that has not yet been successfully managed [10]. Previous studies on epidemics have shown that frontline nurses have different levels of burnout. In the Hu et al [11]. study, about half of frontline nurses reported moderate and high work burnout, and the emotional exhaustion rate was 60.5%, depersonalization was 42.3%, and personal accomplishment was 60.6%. In the Zhang et al. [12] study, 78.5 and 92.5% of nurses presented mild levels of emotional exhaustion and depersonalization, respectively, while participants experienced a severe lack of personal accomplishment. It is related to nurses’ job satisfaction and patients’ outcome [13].

Previous studies showed that burnout factors mainly contain sociodemographic and work-related factors. Sociodemographic factors mentioned in previous studies include sex, age, marital status [14, 15], education [16, 17], having children, living with family [18], personality [19], etc. Work-related factors include employment status [14], work experience [15], work department [20–22], type of shift [14, 15], salary level [23, 24], social support [17, 25, 26], and lack of material resources [25], job stress [16, 25, 27], coping strategies [15, 27], job security and satisfaction [15], education and training [27]. Studies have shown that burnout is related to a range of self-reported physical and psychological stressors, such as anxiety and depression [11], fatigue [28].

However, considering the peculiarity of COVID-19, the level and factors of burnout among nurses at the epicenter are not fully understood, especially concerning different facets of the work environment and concomitant psychological responses, such as anxiety. Therefore, the purpose of this study is to investigate the status of burnout among nurses and its influencing factors during the COVID-19 epidemic to provide intervention decisions for managers.

## Methods

### Design

A multicenter, descriptive, cross-sectional survey was used.

## Participants

The study was conducted from 19 to 25 February 2020, and a self-reported questionnaire was distributed anonymously online to 1011 nurses. Participants have worked in Wuhan tertiary hospitals for at least one week since the epidemic. They were informed of the purpose, and their participation was interpreted as informed consent, and all data were guaranteed confidentiality. Questionnaires with missing values or similar answers for most items were excluded. The final number of valid questionnaires was 885, and the effective response rate was 87.5%.

## Measures

### Social-demographic and work-related variables

The social-demographic and work-related data collection sheets were self-designed and included gender (female, male), age, marital status ( single, married, other), no siblings (yes, no), number of children (0,1,2, ≥ 3), educational level, technical title medical team (Wuhan, others), employment status (regular payroll, personnel agency, contract worker, hiring), work experience (years), current units (fever clinics, isolation ward, ordinary ward), hours of daily work, number of night shifts weekly, dwelling place (hospital dormitory, hotel, home, other), knowledge of COVID-19 (best, better, general, weak), being surrounded by confirmed or suspected medical staff (yes, no).

## Burnout

Burnout was measured with the Maslach Burnout Inventory-General Survey (MBI-GS), developed by Maslach et al [8]. and revised by Li et al. [29]. And its validity has been verified [30]. The 15-item questionnaire contains five questions on emotional exhaustion, four on cynicism, and six on personal accomplishment. Each item is a self-reported question made up of a 7-point scale (0–6 points from "never" to "every day"). The higher score of emotional exhaustion and cynicism is, the higher level of burnout will be. Emotional exhaustion level can be interpreted as mild (0–16), moderate (17–24), or high (≥ 25); cynicism dimension is classified as mild (0–7), moderate (8–11), or high (≥ 12). The score in personal accomplishment is the opposite and is classified as mild (≥ 16), moderate (12–15), or high (0–11) based on item scores [31]. In this study, the Cronbach's a of the MBI-GS was 0.854. The Cronbach's a for three dimensions were 0.957, 0.929, and 0.936, respectively.

## Anxiety

The State-Trait Anxiety Inventory (STAI) was developed by Spielberger et al [32]. And it was revised by Li and Qian [33]. This measure consists of two subscales: State Anxiety Inventory (SAI) and Trait Anxiety Inventory (TAI). The TAI reflects the relatively stable aspect of “anxiety proneness”, while the SAI assesses the current state of anxiety or feeling now [34]. It consists of 40 items, each scored on a 4-point scale (1–4 points), with 20 items for each subscale. The total score is 20–80. The higher the score, the greater the anxiety. It has better reliability and validity [34, 35]. In our study, the Cronbach’s a of SAI was 0.925.

## Statistical analysis

Data analysis was performed using IBM SPSS version 25.0 for windows [36]. Descriptive statistics were used to summarize sample characteristics and outcome variables. Nurses’social-demographic, work-related, and categorical outcome variables were shown as number (N) and percentage (%). Continuous outcome variables were shown as mean and standard deviation (SD). Multivariate Linear Regression analysis was performed to detect independent factors related to anxiety and burnout. The correlation between burnout and anxiety was examined by Pearson correlation. A two-tailed *P* < 0.05 was identified as statistically significant.

## Results

### Social-demographic and work-related situations of participants

The average age of participants was 30.96 (SD = 6.10), aged between 20 and 53. The majority of participants were female (96%), married (61.6%). 71.5% of the participants had no siblings. 42.1% had no children. 86.8% hold a bachelor's degree or above. 69.3% received the junior title. Most were from other regional medical teams (61.6%), and 38.4% were Wuhan's. The majority (65.9%) were contractual workers. During the epidemic, 5.8% worked in the fever clinics, 82.4% in isolation wards, and 11.9% in ordinary wards. Their daily work hours were 5 ~ 8 h (79.1%). 85.6% had one night shift per week at least. Their dwelling places during the epidemic were hospital dormitory (30.2%), hotel (49.8%), home (15.6%), and others (4.4%). Their levels of knowledge of COVID-19 were best (14.6%), better (64.7%), general (20.1%), and weak (0.6%). 61.5% had confirmed or suspected medical staff around themselves. (Table 1.)

**Table 1:** Social-demographic and work-related situations of participants [*N* (%)]

Variables	Mean (SD)	N (%)
Gender		
Male		35(4.0)
Female		850(96.0)
Age	30.96 (6.10)	
Marital status		
Single		326(36.8)
Married		545(61.6)
Divorced/widow		14(1.6)
No siblings		
Yes		252(28.5)
No		633(71.5)
N of Children		
0		373(42.1)
1		404(45.6)
2		100(11.3)
≥ 3		8(0.9)
Education level		
College and below		117(13.2)
Undergraduate		753(85.1)
Master and above		15(1.7)
Technical title		
Junior		613(69.3)
Intermediate		245(27.7)
Deputy Senior and above		27(3.1)
Medical team		
Wuhan		340(38.4)
Others		545(61.6)
Employment status		
Regular payroll		114(12.9)
Personnel agency		113(12.8)
Contract worker		583(65.9)
Hiring		75(8.5)
Work experience, y		
< 5		290(32.8)
5 ~ 10		311(35.1)
11 ~ 20 > 20		203(22.9) 81(9.2)
Current unit		
Fever clinics		51(5.8)
Isolation ward		729(82.4)
Ordinary ward		105(11.9)
Hours of daily work		
≤ 4 5 ~ 8		86(9.7) 700(79.1)
≥ 9		99(11.2)
N of night shifts weekly		
0		127(14.4)
1		120(13.6)
2 ≥ 3		431(48.7) 207(23.4)
Dwelling place		
Hospital dormitory		267(30.2)
Hotel		441(49.8)
Home		138(15.6)
Other		39(4.4)
Knowledge level of COVID-19		
Best		129(14.6)
Better		573(64.7)
General		178(20.1)
Weak		5(0.6)
Confirmed or suspected medical staff around		
Yes		544(61.5)
No		341(38.5)

## The descriptive statistics of burnout and anxiety scales

The potential range score, mean values, standard deviations (SD), and the lowest and highest scores of the subscales are listed in Table 2. The prevalence of low, moderate, and high emotional exhaustion, cynicism, and personal accomplishment is shown in Table 3.

**Table 2:** The descriptive statistics of burnout and anxiety scales (*n* = 885)

	**Range**	**Lowest score**	**Highest score**	**Mean**	**SD**
SAI	20–80	20	78	45.52	10.50
TAI	20–80	20	78	43.78	9.86
EE	0–30	0	30	11.50	7.51
Cynicism	0–24	0	24	6.02	5.58
PA	0–36	0	36	24.47	9.53

*SD***,** Standard Deviation; *SAI*, State Anxiety Inventory; *TAI*, Trait Anxiety Inventory; *EE*, Emotional exhaustion, *C*, Cynicism, *PA*, Personal accomplishment

**Table 3:** The prevalence of each dimension of burnout scale (*n* = 885, %)

	**Mild**	**moderate**	**high**
EE	685(77.40%)	129(14.58%)	71(8.02%)
C	563(63.62%)	181(20.45%)	141(15.93%)
PA	701(79.21%)	110(12.43%)	74(8.36%)

## Correlation between burnout and anxiety

The linear correlation between burnout scales and the anxiety scores was determined in Table 3. The emotional exhaustion scale presented statistically significant correlations with SAI and TAI scores, and statistically significant relationship was observed between cynicism and SAI and TAI items. Similarly, significant correlations were found between personal accomplishment and SAI and TAI scores. There was a significant positive correlation between emotional exhaustion and cynicism but no significant correlation with personal accomplishment. Cynicism was negatively related to personal accomplishment (Table 4).

**Table 4:** Correlation coefficients between burnout and anxiety (*N* = 885)

	SAI	TAI	**EE**	**C**	**PA**
SAI	1	0.883**	0.553**	0.494**	-0.275**
TAI		1	0.605**	0.547**	-0.319**
EE			1	0.729**	-0.042
C				1	-2.18**
PA					1

*S****AI*** State Anxiety Inventory, *TAI* Trait Anxiety Inventory, *EE* Emotional exhaustion, *C*, Cynicism, *PA*, Personal accomplishment

^**^P < 0.01

## Factors associated with burnout

Multiple linear regression analysis was compiled for each dimension of the burnout scale. For emotional exhaustion, the variables personnel agency (β = -2.01, *P* < 0.01), five years or less working experience (β = -1.75, *P* = 0.001), living in hospital dormitory (β = -1.86, *P *= 0.001), three or more night shifts weekly (β = 2.43, *P* < 0.001), the best (β = -3.24, *P* < 0.001) and better (β = -1.25,* P* < 0.05) level of knowledge of COVID-19, and having confirmed or suspected medical staff around (β = 2.05, *P* < 0.001) were statistically significant predictors. For cynicism, the variables intermediate title (β = 1.21, *P* < 0.01), personnel agency (β = -1.72,* P* < 0.01), working in isolation ward (β = 1.04, *P* < 0.05), living in hospital dormitory (β = -1.31, *P* = 0.001), 3 or more night shifts weekly (β = 2.10, *P* < 0.001), the best level of knowledge of COVID-19 (β = -1.83, *P* < 0.001), and having confirmed or suspected medical staff around (β = 1.71, *P* < 0.001) were predictors. Finally, for personal accomplishment, no siblings (β = -1.73, *P* < 0.05), Wuhan medical team (β = 1.37,* P* < 0.05), living in hotel (β = -1.33, *P* < 0.05), nine or more hours of daily work (β = 3.37, *P* = 0.001), the best (β = 5.67, *P* < 0.001) and better (β = 2.62, *P* = 0.001) level of knowledge of COVID-19, and having confirmed or suspected medical staff around (β = -1.68, *P* = 0.01) were predictors (Table 5).

**Table 5:** Multivariate Linear Regression Analysis with step-wise methods with burnout subscales as dependent variables (*n* = 885)

	**Emotional exhaustion**		**Cynicism**		**Personal accomplishment**	
	**β**	**SE**	**β**	**SE**	**Β**	**SE**
**Social-demographic factors**						
No siblings					-1.73*	0.69
Intermediate title			1.21**	0.40		
Personnel agency	-2.01**	0.74	-1.72**	0.55		
**Work-related factors**						
Work experience < 5 years	-1.75***	0.52				
Wuhan medical team					1.37*	0.69
Isolation ward			1.04*	0.47		
Hospital dormitory	-1.86***	0.54	-1.31***	0.40		
Hotel					-1.33*	0.66
≥ 3 night shifts weekly	2.43***	0.57	2.10***	0.42		
≥ 9 h of daily work					3.37***	0.99
Knowledge level of COVID-19 Best Better	-3.24*** -1.25*	0.84 0.61	-1.83***	0.51	5.67*** 2.62***	1.07 0.78
confirmed or suspected medical staff around	2.05***	0.50	1.71***	0.37	-1.68**	0.65

^***^P < 0.001,**P < 0.01,*P < 0.05

*Β*, Regression Coefficient; *SE*, Standard Error of the Estimate

## Discussion

### Anxiety and burnout

The results showed that nurses experienced a high level of anxiety during the COVID-19 epidemic. Compared to standard values [37], there were statistical differences on both SAI (*U* = 18.54, *P* < 0.001) and TAI (*U* = 7.46, *P* < 0.001). A previous study found that the values of 31 to 49 indicated moderate anxiety [38]. It noted that 90.66% of participants had moderate or higher anxiety in our study.

In this study, the burnout level of nurses tended to be mild to moderate. Ge's study [39], a survey among Chinese community health workers, showed that emotional exhaustion and cynicism were more severe, with no statistically significant differences in personal achievement. In other studies, the results were 9.7(± 7.1), 6.7(± 5.3), and 28.2(± 5.5) for the Norwegian population [40], and the results for Korea were 18.9(± 7.1), 11.1(± 4.9), and 27.4(± 6.4) [30]. Compared with the two foreign studies, this study scored higher on emotional exhaustion than the Norwegians but lower than Korean. The level of cynicism and personal accomplishment was lower than theirs.

This study reports that more anxiety was associated with higher emotional exhaustion, cynicism, and lower personal accomplishment, consistent with other research findings [22]. This result highlights the importance of early detection of the psychological status of health care workers and of positive interventions to prevent or reduce psychological problems.

This study also showed that the higher the level of emotional exhaustion, the greater the cynicism, and the lower the personal accomplishment, confirming a previous study [41]. Nurses still working during the epidemic faced tremendous pressure, high demands, and volatility. They often deal with the continued suffering of patients, medical futility, the end of life, and ethical issues [42].

### Factors with Burnout

This study analyzed nurses' burnout during the COVID-19 epidemic from social-demographic characteristics and work-related factors based on different dimensions of the burnout scale.

### Social-demographic characteristics

In the current study, nurses who did not have siblings had lower personal accomplishment than those who had siblings. It may be because most nurses without siblings are very young, and they work for shorter terms and lack work experience. In addition, the condition of patients infected with COVID-19 sometimes changes rapidly, but the care function may be limited [22]. On the other hand, siblings reflect social and emotional support [43]. Intimate partner support may increase enthusiasm for work to some extent.

This study showed that nurses with intermediate title had a higher cynicism level. Domestic research showed that nurses with lower technical title were more burnout [44]. This may be because participants with intermediate title mostly had their own families and children in this study. They worry that they may be virus carriers and pass it on to their families, so they cannot go home or take after their young children, and they may focus more on their families.

This study found that nurses with personnel agents had lower emotional exhaustion and cynicism, consistent with other studies [45, 46]. Currently, clinical nurses are employed in hospitals, including regular payroll, personnel agency, contracts, and temporary employment, with different salaries, benefits, promotion of functional titles, and career prospects. Wages and welfare are essential life support for clinical nurses. If basic needs are not met, there will be no incentive to motivate nurses' enthusiasm and satisfaction, especially during the epidemic. On 23 February 2020, the national government put forward a series of measures, such as increasing remuneration packages, implementing first-line personnel life security, strengthening personal protection, etc. [47], to care for healthcare workers to encourage them to maintain total energy concentrate on fighting the epidemic.

### Work-related situations

This study shows that nurses with five or fewer work experiences had lower emotional exhaustion than other longer work experiences. The result differs from a previous study of SARS [48], which showed that nurses with shorter tenure were more likely to consider quitting**.** Work experience is often a contributing factor to burnout [22, 49]. Perhaps, the reason why it was not in line with the literature is that most of the participants are "Post-90 s" in this study. Although most were more anxious, they worked still enthusiastically and actively.

Wuhan medical team members had a higher sense of personal accomplishment. Nurses in the epicenter, Wuhan, as native warriors, had more responsibility and obligation to fight the epidemic and protect her homeland.

Current research suggested that working in isolation wards is a negative factor of cynicism. The reason may be that nurses have to bear their own physical needs in isolation wards. To save protective materials, they couldn't take off their heavy PPE until 4–6 h at least when they are not allowed to eat, drink, or use the toilet. Meanwhile, they risk infection by providing patients with complicated and trivial care and assistance. However, this study reported that the better or best level of knowledge of COVID-19 is a protective factor of burnout. Previous studies had established that training and education about the etiology of emerging infectious disease and related infection control measures would increase their knowledge, help them take a positive view of their perceived anxiety and personal safety, and reduce their level of burnout [48, 50]. Therefore, training the knowledge and skills of healthcare workers who care for COVID-19 patients is a necessary and positive measure.

This study found that the number of night shifts, especially three or more, affects emotional exhaustion and cynicism, consistent with other studies [22, 51]. In the early stage of the epidemic, staff shortages led to shifting work frequently and placed increased pressure on nurses. However, an exciting finding in this study was that nurses who worked more than 9 h a day had a higher sense of personal accomplishment. Despite their heavy workloads, they had a high level of achievement and satisfaction, which was different from previous studies [12]. During the particular period when the whole nation joined forces to fight against the epidemic, they showed great enthusiasm and dedication, which was in line with the traditional national spirit of the Chinese nation.

During the anti-epidemic period, 80% of nurses lived directly in hospital dormitories and hotels. The study showed that living in hospital dormitories is a protective factor for emotional exhaustion and cynicism while living in hotels had lower personal accomplishment. If they had confirmed or suspected medical staff around themselves, they experienced a higher level of burnout. The findings alerted managers to the safety of medical staff and take protective measures and care for them.

### Implications

Nurses are the backbone of medical and health services, especially in emergencies. Managers and supervisors should pay attention to their job satisfaction, occupational health, and personal well-being and take some measures. Firstly, establish psychological consultation clinics for nurses to understand their needs and mental health status. Secondly, improve the salary as much as possible to reduce nurses' financial pressure, and avoid intensive shift work. Then, provide learning opportunities and focus on the growth and empowerment of junior nurses.

### Limitations

This study adopted a cross-sectional survey, which only knew and analyzed the situation at that time. Longitudinal studies will be attempted to further understand the level of burnout of medical staff as the epidemic develops over time. Then, we used the MBI-GS rather than the MBI-Human Services Survey, which may lead to different levels of nurses’ burnout. Researchers tried to use different versions of the MBI scale to evaluate and compare as much as possible. Participants in this study were selected by convenience sampling. In addition, based on the actual situation and literature review, there may be other influencing factors of burnout besides those involved in this study. Further studies can be conducted to explore additional factors and follow up to pay attention to psychodynamics.

## Conclusion

This study aimed to investigate the levels of anxiety and burnout of nurses working in Wuhan during the COVID-19 epidemic and analyze factors affecting burnout. The findings showed that frontline nurses had severe anxiety and mild to moderate burnout. The influencing factors of burnout were analyzed based on social demography and work-related characteristics, and finally, 12 variables were proposed. It will provide a reference for nursing managers and policymakers, especially considering that the epidemic will be a long process. Local or national longitudinal studies will be further conducted to understand nurses' occupational psychological dynamics. And it is immediate to perform individual or group psychological interventions. Make it possible to establish a stable and harmonious nursing team for challenges in the future.

## Data Availability

The datasets used and/or analysed during the current study available from the corresponding author on reasonable request.

## Abbreviations

COVID-19: Coronavirus disease 2019
MBI-GS: Maslach Burnout Inventory-General Survey
STAI: State-Trait anxiety inventory
SAI: State Anxiety Inventory
TAI: Trait Anxiety Inventory
PPE: Personal protective equipment
